# Modelling the cost-effectiveness of person-centred care for patients with acute coronary syndrome

**DOI:** 10.1007/s10198-020-01230-8

**Published:** 2020-09-07

**Authors:** Laura Pirhonen, Hanna Gyllensten, Andreas Fors, Kristian Bolin

**Affiliations:** 1 Institute of Health and Care Sciences, Sahlgrenska Academy, University of Gothenburg, Box 457, 405 30 Gothenburg, Sweden; 2 Centre for Person-Centred Care (GPCC), University of Gothenburg, Gothenburg, Sweden; 3 Centre for Health Economics (CHEGU), Department of Economics, University of Gothenburg, Gothenburg, Sweden; 4 Närhälsan Research and Development Primary Health Care, Region Västra Götaland, Sweden

**Keywords:** Markov model, Person-centred care, Acute coronary syndrome, Randomized-controlled trial, Mid-term cost-effectiveness, I1 (Health)

## Abstract

**Background:**

Person-centred care has been shown to be cost-effective compared to usual care for several diseases, including acute coronary syndrome, in a short-term time perspective (< 2 years). The cost-effectiveness of person-centred care in a longer time perspective is largely unknown.

**Objectives:**

To estimate the mid-term cost-effectiveness of person-centred care compared to usual care for patients (< 65) with acute coronary syndrome, using a 2-year and a 5-year time perspective.

**Methods:**

The mid-term cost-effectiveness of person-centred care compared to usual care was estimated by projecting the outcomes observed in a randomized-controlled trial together with data from health registers and data from the scientific literature, 3 years beyond the 2-year follow-up, using the developed simulation model. Probabilistic sensitivity analyses were performed using Monte Carlo simulation.

**Results:**

Person-centred care entails lower costs and improved effectiveness as compared to usual care, for a 2-year time and a 5-year perspective. Monte Carlo simulations suggest that the likelihoods of the person-centred care being cost-effective compared to usual care were between 80 and 99% and between 75 and 90% for a 2-year and a 5-year time perspective (using a 500,000 SEK/QALY willingness-to-pay threshold).

**Conclusions:**

Person-centred care was less costly and more effective compared to usual care in a 2-year and a 5-year time perspective for patients with acute coronary syndrome under the age of 65.

**Electronic supplementary material:**

The online version of this article (10.1007/s10198-020-01230-8) contains supplementary material, which is available to authorized users.

## Introduction

Acute coronary syndrome (ACS) is a diagnosis which comprises ST-segment elevation myocardial infarction (MI), non-segment elevation MI, and unstable angina [1]. Mortality is elevated among patients with ACS both during the initial hospitalization and (at least) up to 12 years after discharge. The reported long-term mortality rate after an ACS varies between studies. For example, in a large international study, patients with ACS had a 2-year overall mortality rate of 5.5% [2], while, in a study from New Zealand, the 12-year all-cause mortality rate after an ACS was as high as 52% [3]. ACS is associated with considerable direct costs, both during [4] and directly after hospitalization [5], and at least up to 5 years after discharge [6]. Re-hospitalizations due to cardiovascular conditions are frequent for patients with ACS and is a main contributor to the direct costs associated with the disease [5]. It has been estimated that up to 30% of patients diagnosed with ACS are re-hospitalized within 6 months after the initial ACS event, and one out of five patients will suffer from an ACS-related event during the first 5 years after the initial hospitalization [7, 8]. Moreover, indirect costs, induced by ACS and associated with workplace absenteeism, premature death, and long-term disability, are among the largest for common health conditions in the working-age population [4, 5, 9]. Furthermore, an ACS event has been shown to have a negative impact on the health-related quality of life of the affected patients, both in a short-term and in a long-term perspective [10–12].

Person-centred care (PCC) aims at delivering healthcare that focuses on the patient as a person with unique needs and resources. More specifically, PCC is “co-produced” by the patient and the healthcare professionals as partners to coordinate and tailor care and recovery to the needs and capabilities of each patient [13]. PCC has been shown to result in beneficial effects for patients with ACS, and it has been shown to be cost-effective, compared to usual care, in a 1-year time perspective for patients under the age of 65 [14] (the majority of people in Sweden exit the workforce at the age of 65 [15]). Moreover, PCC has been found to improve patients’ health-related quality of life [16] and their levels of self-efficacy [17, 18].

Previous studies have found PCC to be cost-effective for patients with ACS after 1 year [14] and to have beneficial, but declining, effects after 2 years for the same patients [19]. A study examining the cost-effectiveness of PCC for patients with head and neck cancer found PCC to dominate usual care after 1 year [20]. For patients with chronic heart failure, a person-centred care intervention resulted in less healthcare costs and more health benefits compared to usual care 3 months after inclusion to the study [21]. Furthermore, PCC given to patients with hip fracture was cost-effective compared to usual care after 18 months [22].

Typically, clinical studies span over time periods between 1 and 2 years. Although short-term effects of interventions are essential to study, policy-makers need information spanning over a longer time period. Health-economic modelling is a useful tool for extrapolating short-term information from clinical trials to a longer time perspective [23, 24]. Perhaps, the most frequently applied modelling approach in health economics involves assuming that outcomes evolve according to a Markov process [24]. Health-economic modelling mimicking the progress of various diseases is an essential component in studies of the cost-effectiveness of specific allocations of resources as compared to competing allocations. The literature on the cost-effectiveness of interventions provided to patients with ACS pertains mostly to comparisons between pharmaceuticals, using Markov model approaches to project outcomes beyond the reach of the clinical information [25–33]. To the best of our knowledge, no PCC cost-effectiveness results have been published based on health-economic modelling projecting outcomes beyond the end-point of available clinical information.

In this paper, a Markov-type model of an intervention provided to patients with ACS is developed and employed in the estimation of the mid-term cost-effectiveness of PCC (compared to usual care) utilizing randomized-controlled clinical primary data, register data and data from the scientific literature. Outcomes are projected from a 2-year to a 5-year period (clinical data are available for a 2-year period). In addition, we perform (1) deterministic sensitivity analyses to identify the threshold values of different model parameters at which PCC becomes cost-effective (for a given willingness-to-pay threshold), and (2) probabilistic sensitivity analyses of the cost-effectiveness measure with respect to the values assumed for the parameters of the simulation model.

## Method and data

### The health-economic model

The characterising features of a Markov model is its lack of memory and the use of mutually exclusive states [24]. The Markov-type model developed in this study projects available 2-year clinical data to a 5-year time perspective and computes cost-effectiveness measures of PCC provided to patients with ACS under the age of 65. The model performs parallel calculations for the two compared populations, distinguishing between three states: remission, relapse, and dead. The model incorporates costs associated with sickness absenteeism from work without considering absenteeism as a separate state (see below).

**Fig. 1:**
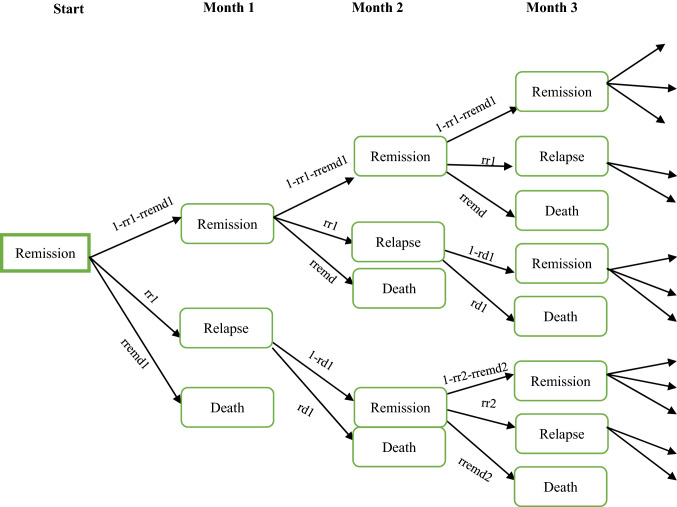
Illustration of the structure of the health-economic simulation model. Figure illustrating the patient pathway in the Markov-type model for the first 3 months. The model continues until 5 years has passed, as indicated by the arrows to the right. *rr1* risk of first relapse, *rremd1* risk of death after first remission, *rd1* risk of death after first relapse, *rr2* risk of death after second relapse, *rremd2* risk of death after second remission

The patient populations start in a state of an ACS event. Thereafter, each population moves to one of the three states. Over the 5-year period, the model accounts for two relapses and three remissions. Once in the third remission, patients are assumed to remain in that state or die. For the first 2 years after the initial ACS event, the risk of relapse changes according to the progress of the disease and differs between treatments, based on available clinical data. Relapse risks for the period between 2 and 5 years are collected from the peer-reviewed literature and do not distinguish between disease progress or treatment. The cycle length of the model is 1 month, i.e., patients move from one state to another after 1 month and, hence, it is assumed that patients remain 1 month in a recurrent event (relapse), from which a transit is made to either the dead state or to remission. Below, in Fig. 1, the structure of the model is illustrated. The simulation model was constructed and all analyses were performed in Excel using its Visual Basic facility.

### Data and intervention

The simulation model was populated with primary data collected from a randomized-controlled trial (RCT) in which PCC was provided for patients with ACS throughout their initial hospitalization and subsequent follow-up in specialized outpatient care and primary care [19, 34], individual-level register data collected from health registers, and with data collected from the scientific literature*.* In the RCT, the PCC intervention was applied according to the framework developed by the University of Gothenburg Centre for Person-Centred Care (GPCC), which underlines the importance of knowing the patient as a person and establising a collaborative and transparent partnership between the patient and healthcare professionals based on ethical principles and mutual respect [13]. The PCC framework comprises of three routines (listening to the patient´s narrative and co-creation of a jointly agreed health plan, shared decision-making, and documenting and sharing the health plan), and aims to involve the patient (often with relatives) as an active partner in the care planning and decision-making process [13, 34]. The simulation model utilized data from patients included in the RCT that were under the age of 65 (*n* = 117).

#### Risks of relapse and mortality

Treatment-specific monthly relapse risks pertaining to the first 2 years were calculated using individual-level register data from the patient register on patients included in the clinical trial (the monthly risk was calculated from the total risk over the 2-year period, defined as the number of relapses divided by the number of patients in, each group). The risk of relapse differed between the two groups, the risk of first relapse was higher for the control group, while the risk of a second relapse was higher for the PCC group. The risk of relapse for the remaining 3 years was assumed to be treatment independent and was calculated using data from Jernberg et al. [35]. Monthly health-state specific, but non-treatment specific, mortality risks were calculated separately for the time periods $<$ 2 years and 2–5 years after the initial event, based on Santos et al. [36], Shotan et al. [37], Piironen et al. [6], Tini et al. [38], and Fox et al. [39]. A summary of the transition probabilities is provided in Table 1.

**Table 1:** Transition probabilities (risks), and state-related costs and quality-of-life weights, and statistical distributions applied in the Monte Carlo simulations

	Usual care	Person-centred care	Probabilistic sensitivity analysis—probability distributions	Source
Risks				
2 year risks				
Relapse 1	0.0115	0.0093	Beta (*α*, *β*)^a^	Trial
Relapse 2	0.0035	0.0056	Beta (*α*, *β*)^a^	Trial
Death after relapse 1	0.0248	0.0248	Beta (*α*, *β*)^a^	Shotan et al. [37]
Death after relapse 2	0.0248	0.0248	Beta (*α*, *β*)^a^	Shotan et al. [37]
Death after remission 1	0.0106	0.0106	Beta (*α*, *β*)^a^	Santos et al. [36]
Death after remission 2	0.0115	0.0115	Beta (*α*, *β*)^a^	Piironen et al. [6]
Death after remission 3	0.0115	0.0115	Beta (*α*, *β*)^a^	Piironen et al. [6]
5 year risks				
Relapse 1	0.0062	0.0062	Beta (*α*, β)^a^	Jernberg et al. [35]
Relapse 2	0.0062	0.0062	Beta (*α*, β)^a^	Jernberg et al. [35]
Death after relapse 1	0.0037	0.0037	Beta (*α*, *β*)^a^	Fox et al. [39]
Death after relapse 2	0.0037	0.0037	Beta (*α*, *β*)^a^	Fox et al. [39]
Death after remission 1	0.0022	0.0022	Beta (*α*, *β*)^a^	Tini et al. [38]
Death after remission 2	0.0022	0.0022	Beta (*α*, *β*)^a^	Tini et al. [38]
Death after remission 3	0.0022	0.0022	Beta (*α*, *β*)^a^	Tini et al. [38]
Costs				
Costs in relapse 1	45,307	39,824	Log-normal (*μ*, *σ*^2^)^b^	Trial
Costs in relapse 2	45307	39,824	Log-normal (*μ*, *σ*^2^)^b^	Trial
Costs in remission 1	2176	2223	Log-normal (*μ*, *σ*^2^)^b^	Trial
Costs in remission 2	2176	2223	Log-normal (*μ*, *σ*^2^)^b^	Trial
Costs in remission 3	2176	2223	Log-normal (μ, *σ*^2^)^b^	Trial
Indirect costs (per month) from sick leave	9349	8250	Log-normal (*μ*, *σ*^2^)^b^	Trial and Statistics Sweden
Average gross income per month^c^	46,400	46,400		Statistics Sweden
Quality-of-life weights (QoL)				
QoL weights relapse 1	0.67	0.67	Beta (*α*, *β*)^a^	Matza et al. [43]
QoL weights in relapse 2	0.67	0.67	Beta (*α*, *β*)^a^	Matza et al. [43]
QoL weights in remission 1	0.82	0.82	Beta (*α*, *β*)^a^	Matza et al. [43]
QoL weights in remission 2	0.82	0.82	Beta (*α*, *β*)^a^	Matza et al. [43]
QoL weights in remission 3	0.82	0.82	Beta (*α*, *β*)^a^	Matza et al. [43]

2 year risks pertain to the period between the first ACS event and 2 years thereafter. 5 year risks pertain to the period from 2 years after the first event and 5 years thereafter

Relapse (ACS-related): ICD codes I20, I21, I22, I25, I5; Relapse 1= ACS event after initial hospitalization for ACS; Relapse 2 = ACS event after relapse 1; indirect costs = all short-term absenteeism from work after initial hospitalization. Costs in relapse = mean costs for inpatient care during an ACS event. Costs in remission = mean costs for all ACS-related primary care, specialized outpatient care and pharmaceuticals (ATC codes: C07, C09, C09, C10, C10AC01, B01AA03, B01AB04, B01AE07, B01AF02, C01AA05, C01BD01, C01CA24, C01DA02, C01DA14, B01AC)

^a^The parameters, $\alpha$ and $\beta$, specifying the distribution are calculated assuming that the deterministic value is the mean of the distribution. The variance was assumed to be 20% of the mean value:

$\alpha =\stackrel{-}{x}\left(\frac{\stackrel{-}{x}(1-\stackrel{-}{x})}{\stackrel{-}{v}}-1\right)$, $\beta =(1-\stackrel{-}{x})\left(\frac{\stackrel{-}{x}(1-\stackrel{-}{x})}{\stackrel{-}{v}}-1\right)$, where $\stackrel{-}{x}$ is the mean and $\stackrel{-}{v}$ is the variance, and $\stackrel{-}{v}<\stackrel{-}{x}(1-\stackrel{-}{x})$

^b^The parameters of the distribution are calculated assuming that the deterministic value is the mean of the non-logarithmized distribution, and that the variance is 20% of the mean value $$\mu = \ln \left[ {\frac{m}{{1 + {\raise0.7ex\hbox{$v$} \!\mathord{\left/ {\vphantom {v {m^{2} }}}\right.\kern-\nulldelimiterspace} \!\lower0.7ex\hbox{${m^{2} }$}}}}} \right]$$ and $${\sigma }^{2}=\mathrm{ln}\left[1+\frac{v}{{m}^{2}}\right]$$, where $$m$$ and $$v$$ are the mean and variance of the non-logarithmized distribution. Analyses were performed using alternative values for the variance of the mean, but this did not change the results qualitatively

^c^The average gross income per month is calculated as the average of gross monthly income for the years 2011–2016 plus labor taxes paid by the employer

Relapse was defined as follows. A patient was assumed to be in relapse when he or she had been hospitalized for an ACS event (defined according to the following ICD codes: ICD20, ICD21, ICD22, ICD25, and ICD50). Remission was defined as not being in relapse.

#### Costs

Cost-effectiveness (base-case) calculations were performed for both a healthcare provider and for a societal perspective [40], including indirect costs due to short-term sick leave and mortality. The healthcare costs associated with relapse and remission were calculated for each treatment alternative using the health-state information collected from the below described registers by linking the data with the patients in the clinical trial.

More specifically, state-dependent healthcare costs were calculated in two steps. First, individual information on healthcare utilization was collected by linking the individuals in the clinical trial, using personal identification numbers, to the information on inpatient- and hospital-based outpatient visits in the National Patient Register (the Swedish National Board of Health and Welfare) and to the information on primary care visits in the regional patient register (VEGA, Region Västra Götaland, Sweden). Individual inpatient and hospital-based outpatient care costs were calculated using year-specific DRG weights associated with each visit, and the cost per DRG unit for 2016 [41]. Primary care costs were calculated using the 2015 per-visit costs associated with type of visit and healthcare professional visited (cost per type of visit collected from Swedish Association of Local Authorities and Regions). Individual pharmaceutical costs were collected from the Swedish National Pharmaceutical register (the Swedish National Board of Health and Welfare).

Second, health-state and treatment-specific (PCC, usual care) healthcare costs associated with relapse were defined as the sum of all (individual) inpatient care costs for ACS events divided by the number of events that were observed in the register data. Consequently, treatment-specific healthcare costs associated with remission were defined as the average individual monthly healthcare cost not associated with inpatient care, where healthcare costs not associated with inpatient care were summarized for each patient and divided by the number of patient months in remission (assuming that a relapse has a duration of 1 month). Similarly, treatment-specific ACS-related pharmaceutical costs were calculated as the ratio between total (individual) pharmaceutical costs induced by heart-related conditions (the ATC codes of the included pharmaceutical are listed in Supplementary material) divided by the number of individuals and by the number of covered months not in relapse. The costs associated with relapse and remission were assumed to be independent of the disease progression and of time.

Sickness absenteeism from work was not included as a state in the simulation model (due to data limitations) and, hence, indirect costs associated with absenteeism were included as individual treatment-specific average monthly costs, calculated as total (individual) absenteeism costs divided by the number of patient months. Information about temporary sickness absenteeism from work was collected by linking each patient in the clinical trial to the information registered by the Swedish Social Insurance Agency. This information comprises information about reimbursement paid for work absenteeism exceeding 14 days in each sickness spell. The total number of days of absenteeism was obtained by adding 14 days to each sickness spell. We had no information about underlying diagnoses causing the absence from work and, hence, we assumed that all registered days of absenteeism were caused by the ACS condition. Permanent sickness absenteeism was included for those patients who went into permanent sick leave after their first ACS event. Indirect costs for individuals who were already in permanent sick leave before their first ACS event were not included. Then, indirect costs were calculated employing the human-capital method [42] where the per-day value of production was calculated as the average monthly gross income (an average of gross monthly income 2011–2016), corrected for labor taxes paid by the employer, and divided by the average number of working days per month in Sweden for those below the age of 65 (20 days). Information on average monthly income was collected from Statistics Sweden. Monthly costs related to sickness absenteeism were assumed to be the same for the period beyond 2 years. In contrast, since dead is a state in the simulation model, mortality-related indirect costs were included and estimated as the value of productivity losses accruing up to 5 years after baseline (employing the human-capital method).

The base-case cost-effectiveness calculations assumed a zero incremental cost associated with delivering PCC for each healthcare visit. The rationale for this is that PCC does not involve amounts of tangible resources different from what usual care requires. There may, however, be an introductory fixed cost related to PCC. This cost will be averaged over a large and increasing number of patients, effectively driving the per-patient cost to zero. The developed simulation model comprises facilities for incorporating both a fixed and a variable treatment cost. Unit costs used in the simulation model are summarized in Table 1.

#### Quality-of-life weights

The outcome measure used in our calculations was quality-adjusted life years (QALYs). The clinical data did not permit the calculation of state-dependent quality-of-life weights, due to an indeterminacy regarding the point in time at which the respondents filled out the questionnaires with which the follow-up information was collected. This means that the healthcare events observed in the register data could not be appropriately associated with the reported quality of life. Thus, all quality-of-life weights were collected from the peer-reviewed literature (Matza et al. [43]). Utility weights were assumed to be independent of disease progression and time (see Table 1).

### Discounting

Costs and quality-adjusted life years were discounted at 3%, in the base -case calculations.

### Sensitivity analysis

We performed unit-variable deterministic sensitivity analysis with respect to (1) discount rates for costs and effects (0% and 5%), (2) the risk of a first relapse (during the first 2 years after the initial ACS event), (3) the risk of a second relapse (during the first 2 years after the initial ACS event), (4) monthly indirect costs due to sickness absenteeism, (5) healthcare costs associated with remission, and (6) healthcare costs associated with relapse. In the analyses pertaining to parameters (2)–(6), the calculations were performed for multiple values in the group receiving PCC ranging from, in each case, the base-case value to a value yielding an incremental cost-effectiveness ratio above the adopted willingness-to-pay threshold. The results of these analyses illustrate the sensitivity of the incremental cost-effectiveness ratio to changes in each specific parameter value in a more comprehensive way than what is achieved in more orthodox unilateral deterministic sensitivity analyses, and provide ranges of the parameter values for which the intervention is cost-effective (for a specific willingness-to-pay threshold). Moreover, probabilistic sensitivity analyses were performed with respect to risks, quality-of-life weights, and costs. The probabilistic sensitivity analysis was performed using Monte Carlo simulation [44]. Utilities and risks were modelled using the beta distribution, while costs were modelled using the log-normal distribution. Using these specifications, Monte Carlo simulations with 1000 random draws were performed. The results were presented plotting (1) the incremental cost and effect (QALYs) pairs in the cost-effectiveness plane, and (2) the shares of the random incremental cost-effectiveness ratios falling below a range of specific values (cost-effectiveness acceptability curve) [45, 46], using a willingness-to-pay threshold of SEK 500,000 per QALY [47, 48]. The included variables and the associated assumed statistical distributions, and their specifications, are reported in Table 1.

## Results

### Base-case analysis

In Table 2, the base-case results and the results from the first part of the deterministic sensitivity analysis (with respect to discount rate for costs and effects) are reported. The number of incremental quality-adjusted life years was estimated at 1.04 and 2.70 for the 2-year and the 5-year time perspective (per 1000 patients), while the number of incremental life years was estimated at about 3 (for the 5-year time perspective). PCC was also associated with lower direct and indirect costs, for both time perspectives, i.e., PCC dominates usual care in both time perspectives. The resulting incremental cost-effectiveness ratio for the 5-year time perspective was estimated at SEK-82,292 per QALY (all costs included).

**Table 2:** Base-case cost-effectiveness results, per 1000 patients, for a 2-year and a 5-year time perspective

	Incremental effect (QALYs)	Increment cost (SEK)	Cost per QALY gained (ICER)
2 years			
Including all costs (total costs)^a^	1.04	− 158,792	− 152,764
Including indirect costs due to sickness absenteeism and direct costs	1.04	− 124,245	− 119,529
Including only direct costs	1.04	− 122,028	− 117,396
5 years			
Including all costs (total costs)^a^	2.70	− 222,314	− 82,292
Including indirect costs due to sickness absenteeism and direct costs	2.70	− 93,484	− 34,604
Including only direct costs	2.70	− 88,308	− 32,688
Deterministic sensitivity analysis			
2 years			
Effects and costs 0% discount rate	1.08	− 164,040	− 151,952
Effects and costs 5% discount rate	1.01	− 155,418	− 153,307
Effects 0% and costs 5% discount rate	1.08	− 155,418	− 143,966
Effects 5% and costs 0% discount rate	1.01	− 164,040	− 161,812
5 years			
Effects and costs 0% discount rate	2.93	− 234,721	− 80,097
Effects and costs 5% discount rate	2.56	− 214,605	− 83,778
Effects 0% and costs 5% discount rate	2.93	− 214,605	− 73,232
Effects 5% and costs 0% discount rate	2.56	− 234,721	− 91,631

Person-centred care compared to usual care. Results are reported including all costs, excluding mortality-related costs, and including direct costs only

*SEK* Swedish krona, *QALY* Quality-adjusted life years, *ICER* Incremental cost-effectiveness ratio

^a^Total cost = direct costs, indirect costs due to short-term sickness absenteeism and indirect costs due to productivity losses due to mortality

The corresponding 5-year incremental cost-effectiveness ratios excluding, first, mortality-related costs and then all indirect costs were estimated at SEK-34,604 and SEK-32,688, per quality-adjusted life year.

### Deterministic sensitivity analysis

The results obtained from the first part of the deterministic sensitivity analysis are presented in Table 2. The results from the remaining sensitivity analysis are presented in the Supplementary material, figures S9–S13. In summary, using a cost-effectiveness ratio of SEK 500,000 per additional QALY as the threshold, the following parameter ranges (in the intervention group) used in the PCC arm resulted in incremental cost-effectiveness ratios below the threshold (1) the risk of first relapse (during the first 2 years after the initial ACS event), from 0.925% (base case) to 1.13%; (2) the risk of second relapse (during the first 2 years after the initial ACS event), from 0.56% (base case) to 5.86%; (3) indirect costs due to sickness absenteeism from SEK 8250 (base case) to SEK 342,290 per month; (4) the healthcare costs associated with being in remission, from SEK 2223 (base case) to SEK 2741 per month; and (5) the healthcare costs associated with relapse, from SEK 39,824 (base case) to SEK 107,302 per month.

### Probabilistic sensitivity analysis

**Fig. 2:**
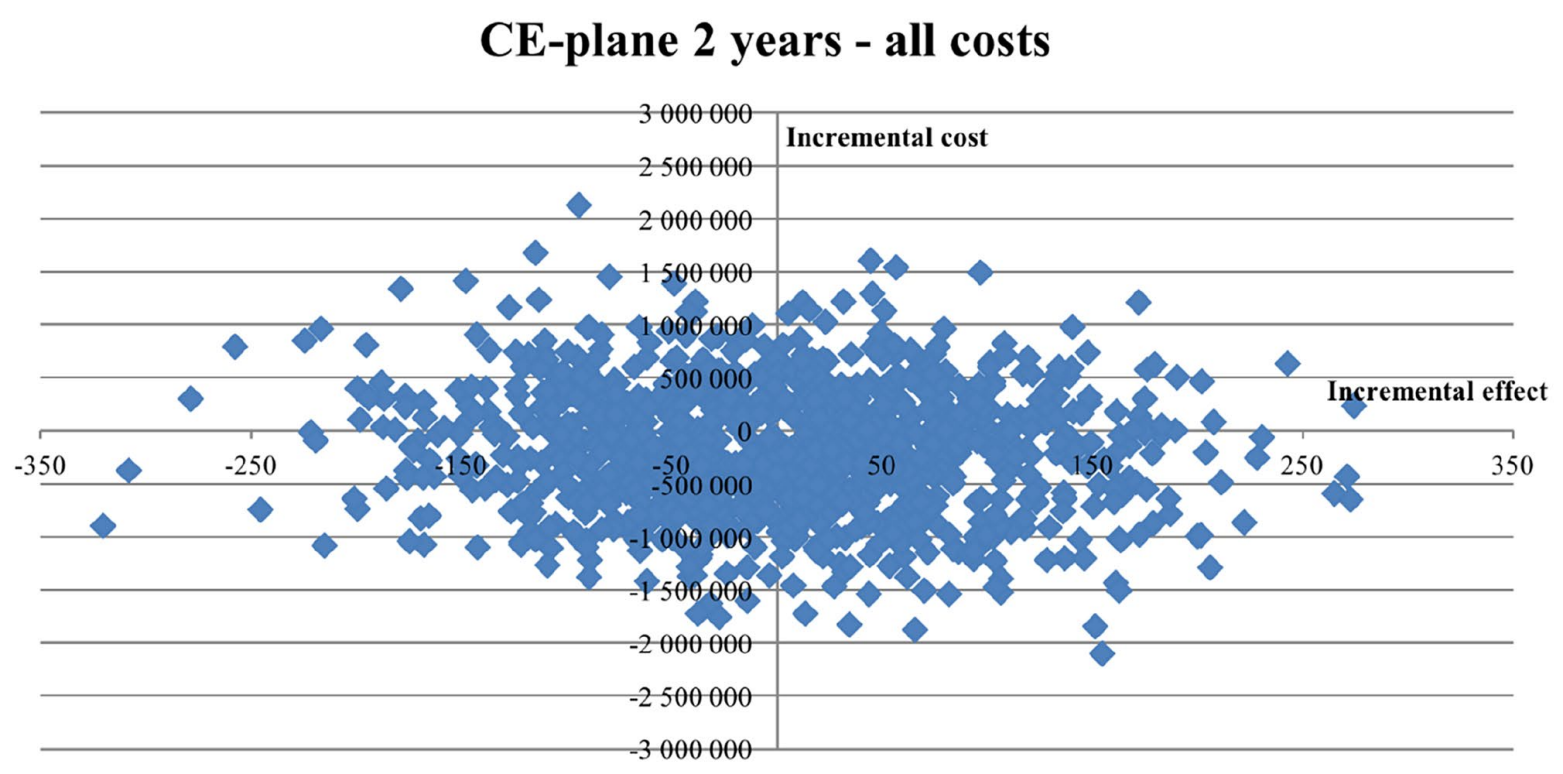
Cost-effectiveness plane (CE plane), illustrating the incremental cost-effectiveness pairs resulting from the 2-year time perspective Monte Carlo simulation. A societal perspective (including all costs). 1000 random draws

**Fig. 3:**
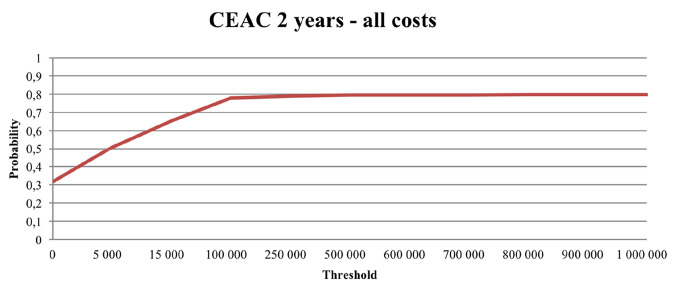
Cost-effectiveness acceptability curve (CEAC) illustrating the likelihood that the incremental cost-effectiveness ratio falls below a given threshold. A 2-year time perspective and a societal perspective (including all costs)

**Fig. 4:**
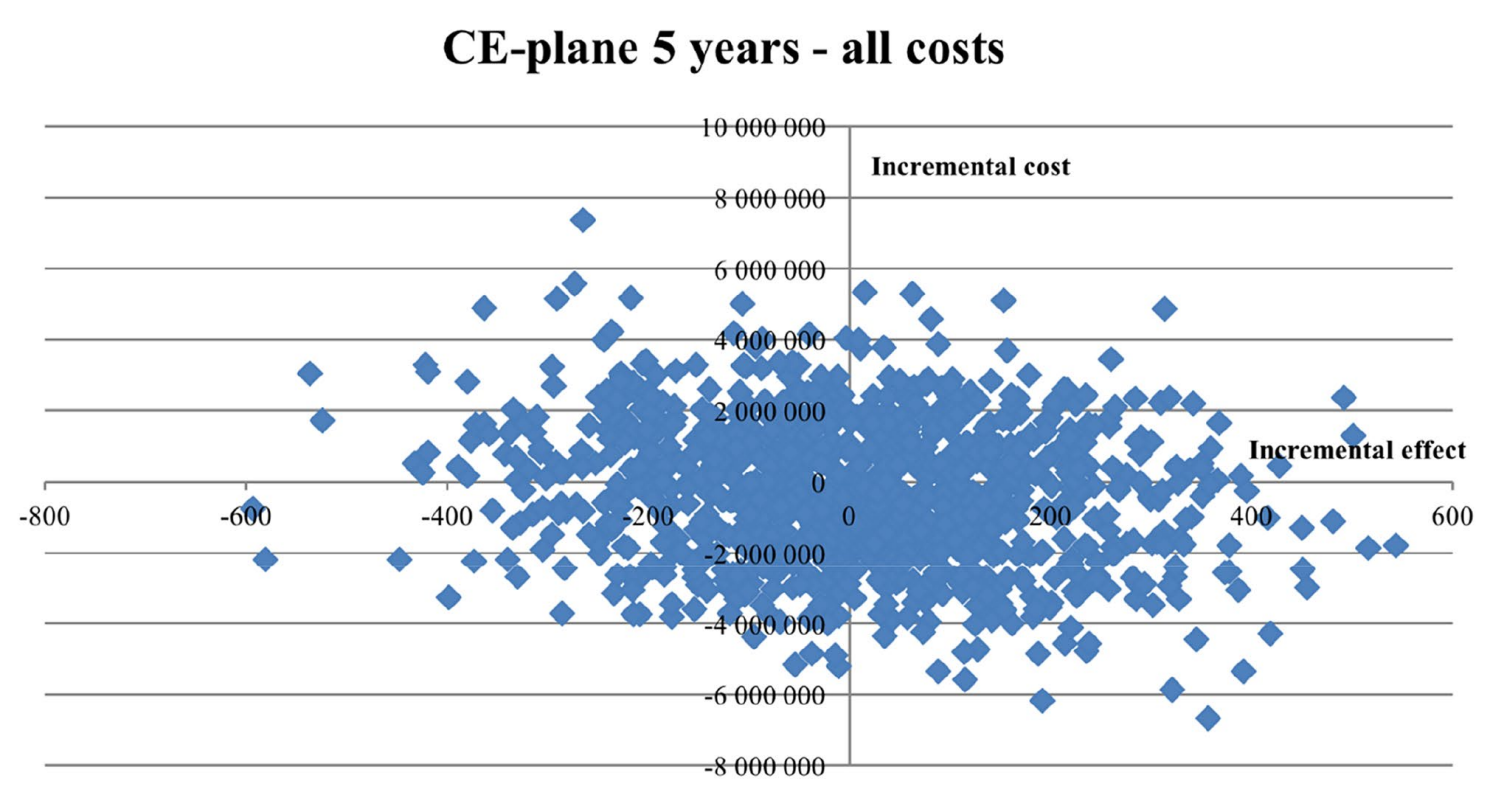
Cost-effectiveness plane (CE plane), illustrating the incremental cost-effectiveness pairs resulting from the 5-year time perspective Monte Carlo simulation. A societal perspective (including all costs). 1000 random draws

**Fig. 5:**
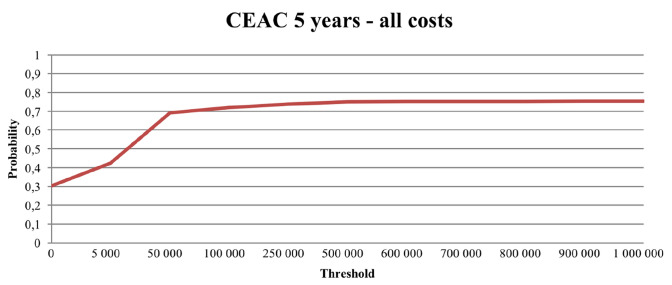
Cost-effectiveness acceptability curve (CEAC) illustrating the likelihood that the incremental cost-effectiveness ratio falls below a given threshold. A 5-year time perspective and a societal perspective (including all costs)

The probabilistic sensitivity results are reported in Figs. 2, 3, 4, and 5. The Monte Carlo simulations suggest that the likelihood that PCC is cost-effective compared to usual care, for a 2-year time perspective, is between 80 and 99%, depending on what cost components that are included. Similarly, for a 5-year time perspective, the likelihood was estimated in the range 75–90%. The results from the corresponding analyses without mortality-related productivity losses and without indirect costs are reported in the Supplementary material (Figures S1–S8).

## Discussion

In this paper, the cost-effectiveness of PCC as compared to usual care provided to patients with ACS was calculated using a health-economic model projecting treatment outcomes and associated costs beyond the reach of available clinical data. In summary, PCC was found to dominate usual care both when applying a 2-year time perspective and a 5-year time perspective. Probabilistic sensitivity analyses suggest that the likelihoods of PCC being cost-effective for the two time perspectives were relatively high (80–99% and 75–90%, respectively). Productivity losses due to mortality were included in the calculation for the covered 5-year period only. Thus, since the number of life years is lower in the comparator arm, our estimates provide a conservative measure of the true cost-effectiveness.

A within-trial cost-effectiveness analysis has previously been employed for the intervention modelled in this paper. Results showed that the intervention was cost-effective compared to usual care for patients under the age of 65 for a 1-year time perspective [14]. To the best of our knowledge, this is the first study in which the cost-effectiveness of PCC provided to patients with ACS has been estimated for a time perspective beyond the reach of available clinical data by health-economic modelling. However, the long-term cost-effectiveness of patient-centred care has been studied, by projecting outcomes for a longer time perspective than covered by clinical data, for other therapeutic areas, for example, type 2 diabetes and heart failure [47–50].

The health-economic simulation model developed has been structured so as to appropriately mimic a broad range of treatments provided to patients with ACS. Thus, the model is potentially applicable in future research in this area, including studies of PCC-based interventions. Previous studies on interventions for patients with ACS have mainly studied interventions pertaining to pharmaceuticals or medical procedures, not complex interventions, such as PCC. Models extrapolating results from trials designed for patients with ACS often model states such as myocardial infarction, post myocardial infarction, and post-stroke [25, 27, 30, 32, 49–51]. Our model is similar to other models which extrapolate results from trials for patients with ACS except for the fact that myocardial infarction is integrated in the ACS state and, hence, is not considered a separate state in our model. In fact, states including frequent co-morbidities are not included in our model. Moreover, the clinical trial from which data for this study were collected included patients with ACS and did not differentiate between different conditions. Nevertheless, we consider our model to be a useful tool for analysing the mid-term cost-effectiveness of new ACS interventions.

Naturally, all cost-effectiveness calculations are afflicted by uncertainty, even when the calculations make use of primary data. This problem is aggravated by the extension of the time horizon beyond the point in time for which clinical data are available. To some extent, this problem can be mitigated by sensitivity analyses. We did perform extensive deterministic sensitivity analyses with respect to specific parameters, as well as probabilistic sensitivity analyses. All results obtained suggest that PCC is cost-effective, as compared to usual care, when provided to patients with ACS, both in a short-term and in a mid-term perspective. More precisely, PCC was found to be the cost-effective alternative for a rather broad range of values from the risk of a first relapse to healthcare costs associated with remission and relapse. Assuming that cost-effectiveness is a prerequisite for the implementation of a given intervention, these results provide a benchmark for the ranges within which this risk and costs need to be confined to achieve cost-effectiveness. Needless to say, our results in this respect are conditioned on the particular parametrization of our simulation model. However, a different context may readily be examined by a re-parametrization of the model.

It is in order to mention some caveats. First, the indeterminacy of the clinical data regarding the exact point in time at which information was provided did not allow us to tie the quality-of-life weights reported by the respondents to each health state. Instead, state-dependent weights were collected from the literature, and were assumed to be the same in both treatment arms. Assuming that PCC has at least the same (positive) effect as usual care, our cost-effectiveness measures are conservative in this respect, as well. Second, we assumed that all transition probabilities are the same across the two treatment arms beyond 2 years, due to the lack of clinical trial data longer than 2 years. Again, assuming that PCC produces at least as beneficial outcomes as usual care in the longer time perspective (> 2 years), the cost-effectiveness measures derived constitute an upper boundary on the incremental cost-effectiveness ratios.

## Conclusions

In this paper, we studied the short- and mid-term cost-effectiveness of a PCC intervention provided to patients with ACS, under the age of 65. PCC resulted in less costs and larger health effects compared to usual care, in a 2-year time perspective. These results were qualitatively the same for the 5-year perspective. This is the first paper to investigate the mid-term (> 2 years) cost-effectiveness of PCC by health-economic modelling, for any disease context.

## Electronic supplementary material

Below is the link to the electronic supplementary material.

Supplementary file1 (DOCX 1780 kb)
