# Supplementary material for: Modelling the cost-effectiveness of person-centred care for patients with acute coronary syndrome
Source: Eur J Health Econ. 2020 Sep 7;21(9):1317–27. doi: 10.1007/s10198-020-01230-8 (PMC7581585; doi:10.1007/s10198-020-01230-8)

**Electronic supplementary material**

*The clinical trial*

During the trial, the patients in the intervention group received person-centred care in addition to usual cardiac care [50] during hospitalization, outpatient care and primary care. The control group received usual cardiac care alone at all three healthcare levels. Patients were randomized to either the intervention or the control group between June 2011 and February 2014. Patients were recruited from two hospitals in Gothenburg, Sweden. Inclusion criteria at the time of randomization were: 1) age under 75 years, and 2) hospitalized for ACS (ICD-10 I21, I20.0 or I20.9). Exclusion criteria at the time of randomization were: 1) expected survival less than 1 year; 2) severe disability; 3) ongoing alcohol and/or drug abuse; 4) planned heart surgery; 5) no current address; 6) attendance to private primary care; 7) participation in a conflicting study [33]. Data on disease-specific and socio-economic characteristics were distributed at baseline (during randomization), 4 weeks, 8 weeks, 6 months, 1 year and 2 years after randomization. The collected trial-data was complemented with register data on individual healthcare utilization, prescribed drugs, sick leave and cause of death for both the intervention and control group.

*ATC-codes used for heart-related pharmaceuticals*

C07, C09, C09, C10, C10AC01, B01AA03, B01AB04, B01AE07, B01AF02, C01AA05, C01BD01, C01CA24, C01DA02, C01DA14, B01AC.

*Alternative probabilistic sensitivity analyses*

The results from probabilistic sensitivity analyses excluding mortality-related indirect costs and for direct costs only are illustrated in Figures S1-S8.

**Fig. S1** Cost-effectiveness plane (CE-plane), illustrating the incremental cost-effectiveness pairs resulting from the two-year time perspective Monte-Carlo simulation. A societal perspective (excluding productivity losses due to mortality). 1 000 random draws


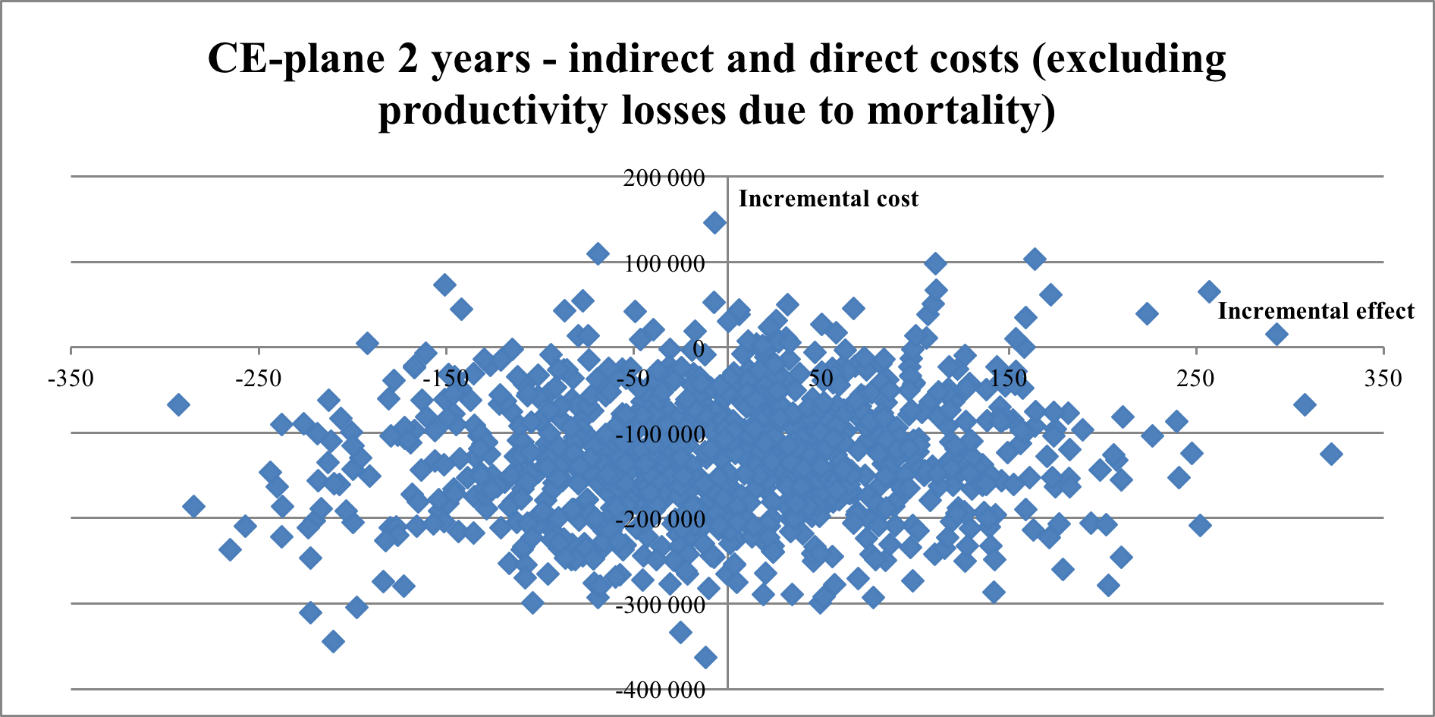


**Fig. S2** Cost-effectiveness acceptability curve (CEAC) illustrating the likelihood that the incremental cost-effectiveness ratio falls below a given threshold. A two-year time perspective and a societal perspective (excluding productivity losses due to mortality)


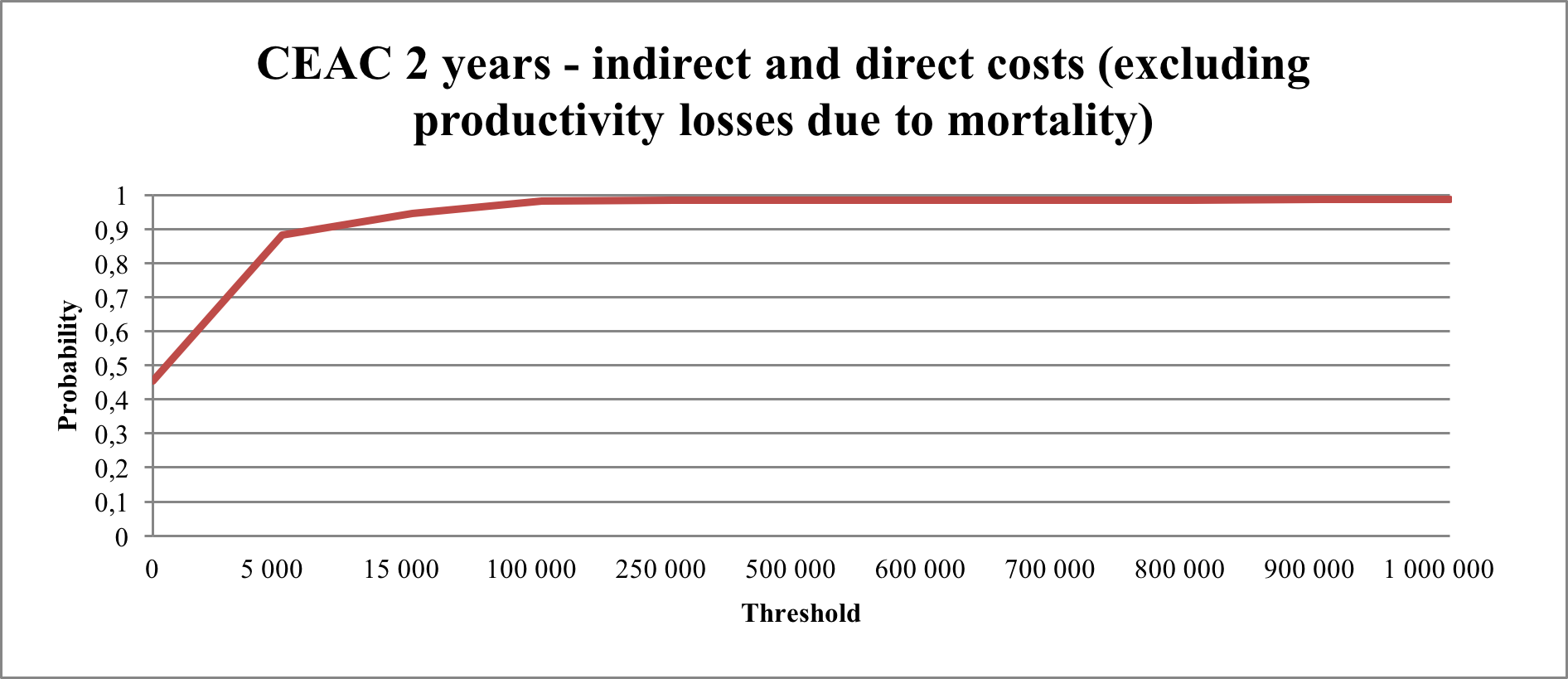


**Fig. S3** Cost-effectiveness plane (CE-plane), illustrating the incremental cost-effectiveness pairs resulting from the five-year time perspective Monte-Carlo simulation. A societal perspective (excluding productivity losses due to mortality). 1 000 random draws


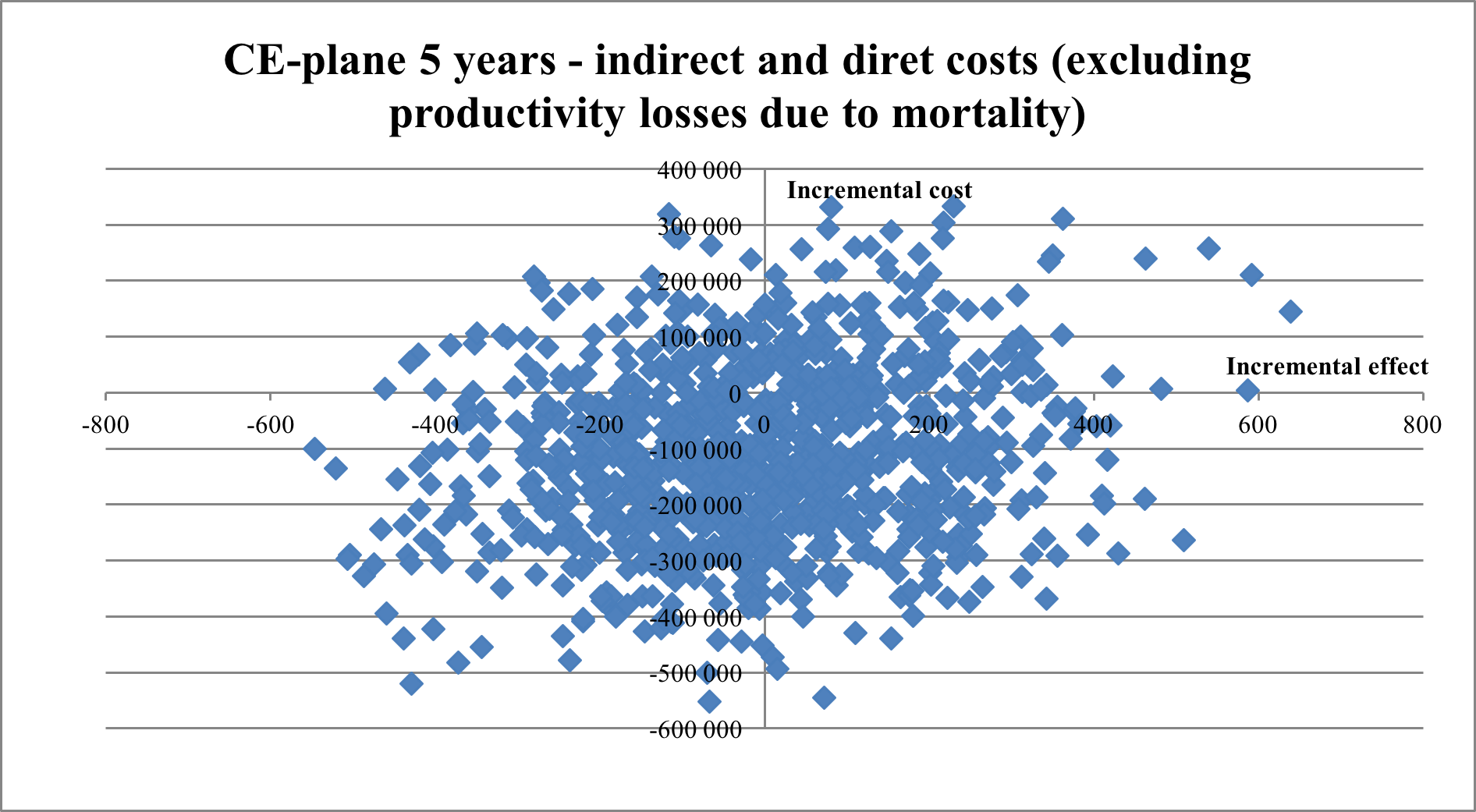


**Fig. S4** Cost-effectiveness acceptability curve (CEAC) illustrating the likelihood that the incremental cost-effectiveness ratio falls below a given threshold. A five-year time perspective and a societal perspective (excluding productivity losses due to mortality)


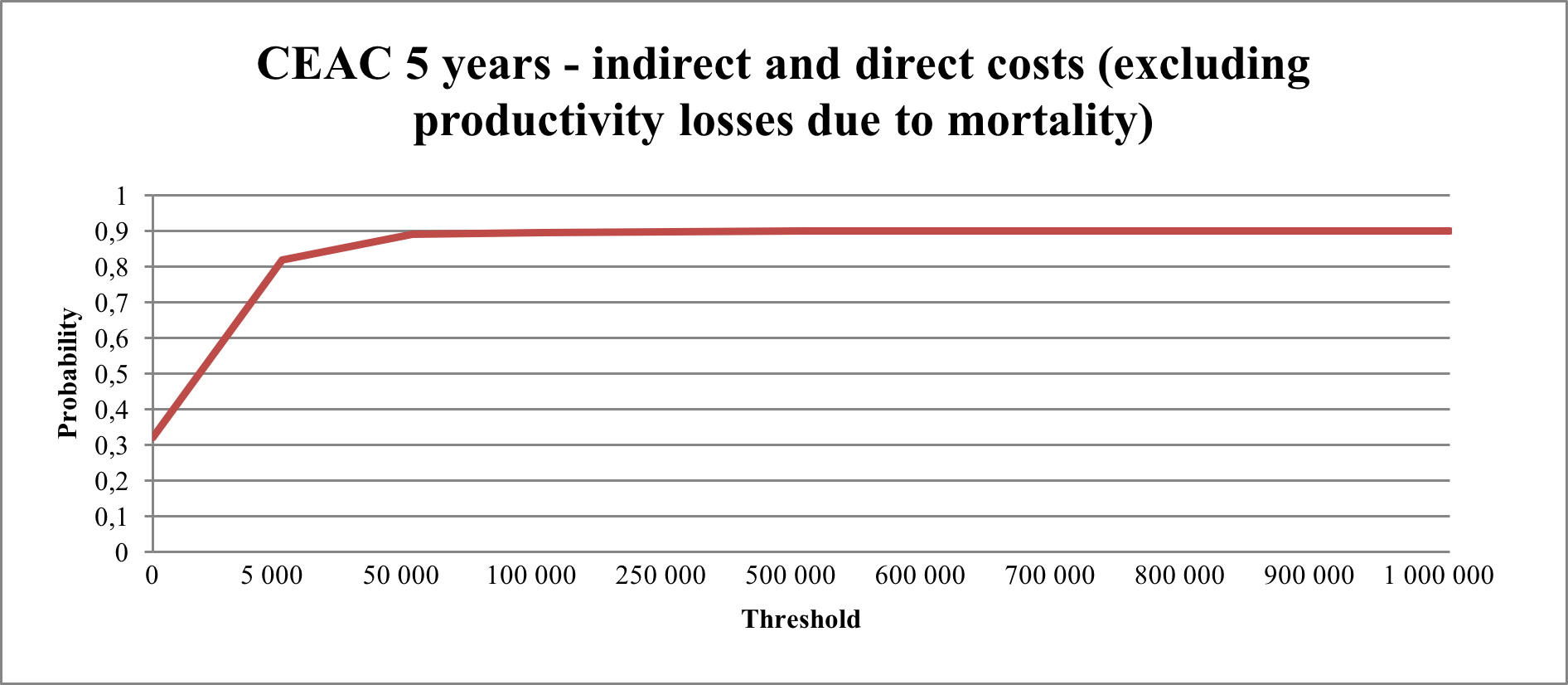


**Fig. S5** Cost-effectiveness plane (CE-plane), illustrating the incremental cost-effectiveness pairs resulting from the two-year time perspective Monte-Carlo simulation. A healthcare perspective. 1 000 random draws


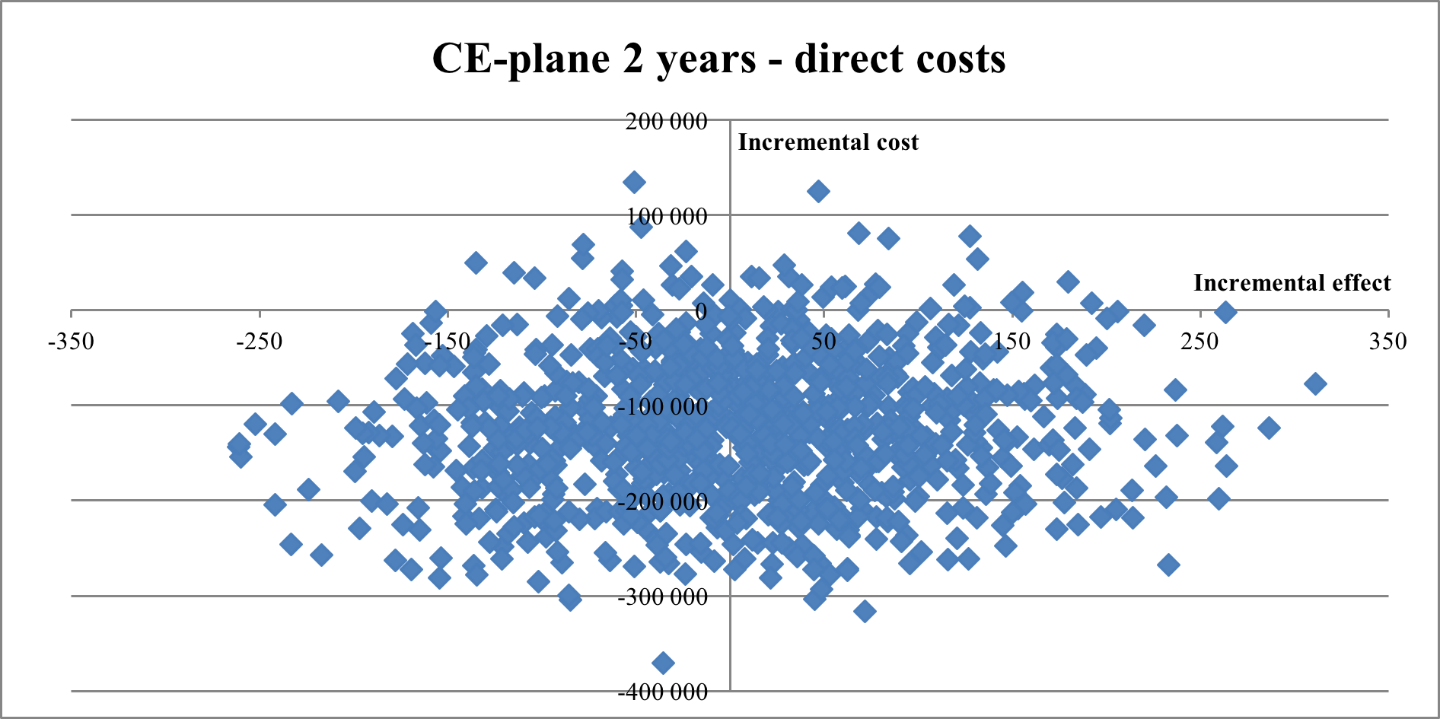


**Fig. S6** Cost-effectiveness acceptability curve (CEAC) illustrating the likelihood that the incremental cost-effectiveness ratio falls below a given threshold. A two-year time perspective and healthcare perspective


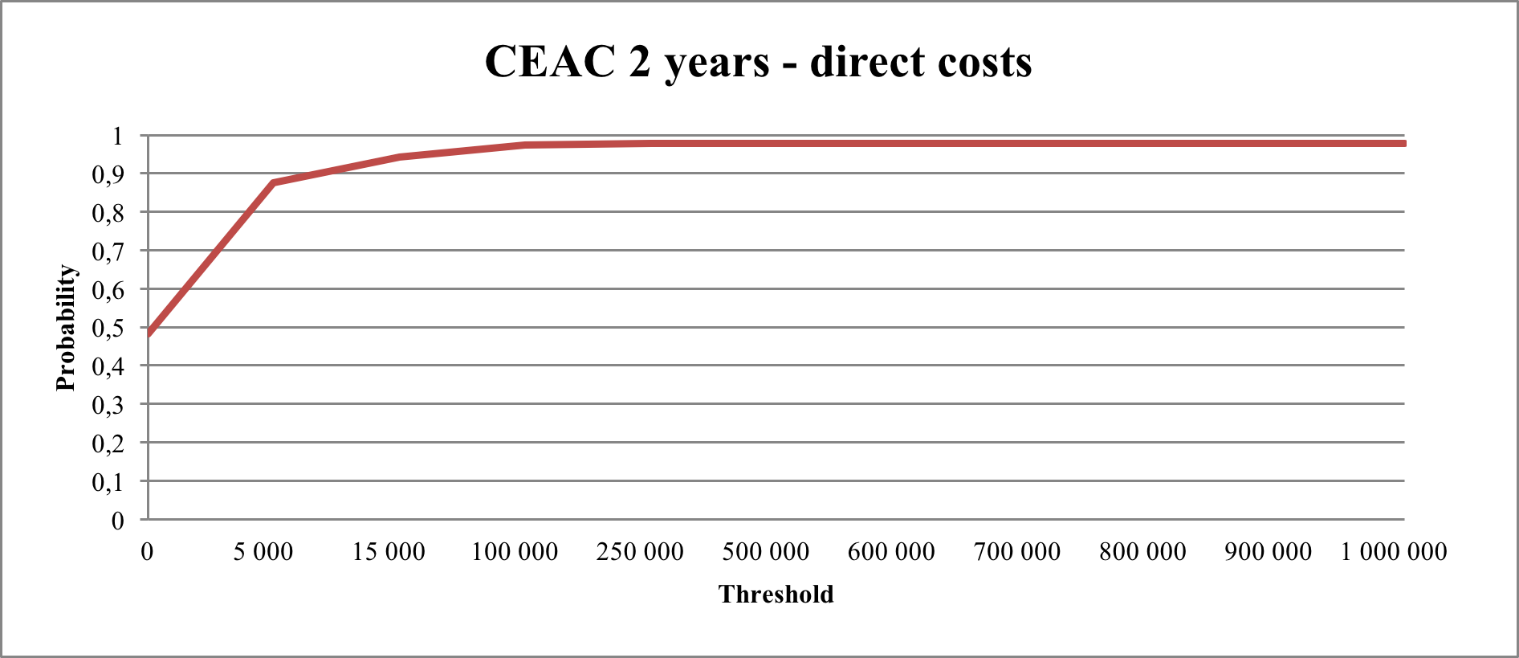


**Fig. S7** Cost-effectiveness plane (CE-plane), illustrating the incremental cost-effectiveness pairs resulting from the five-year time perspective Monte-Carlo simulation. A healthcare perspective. 1 000 random draws


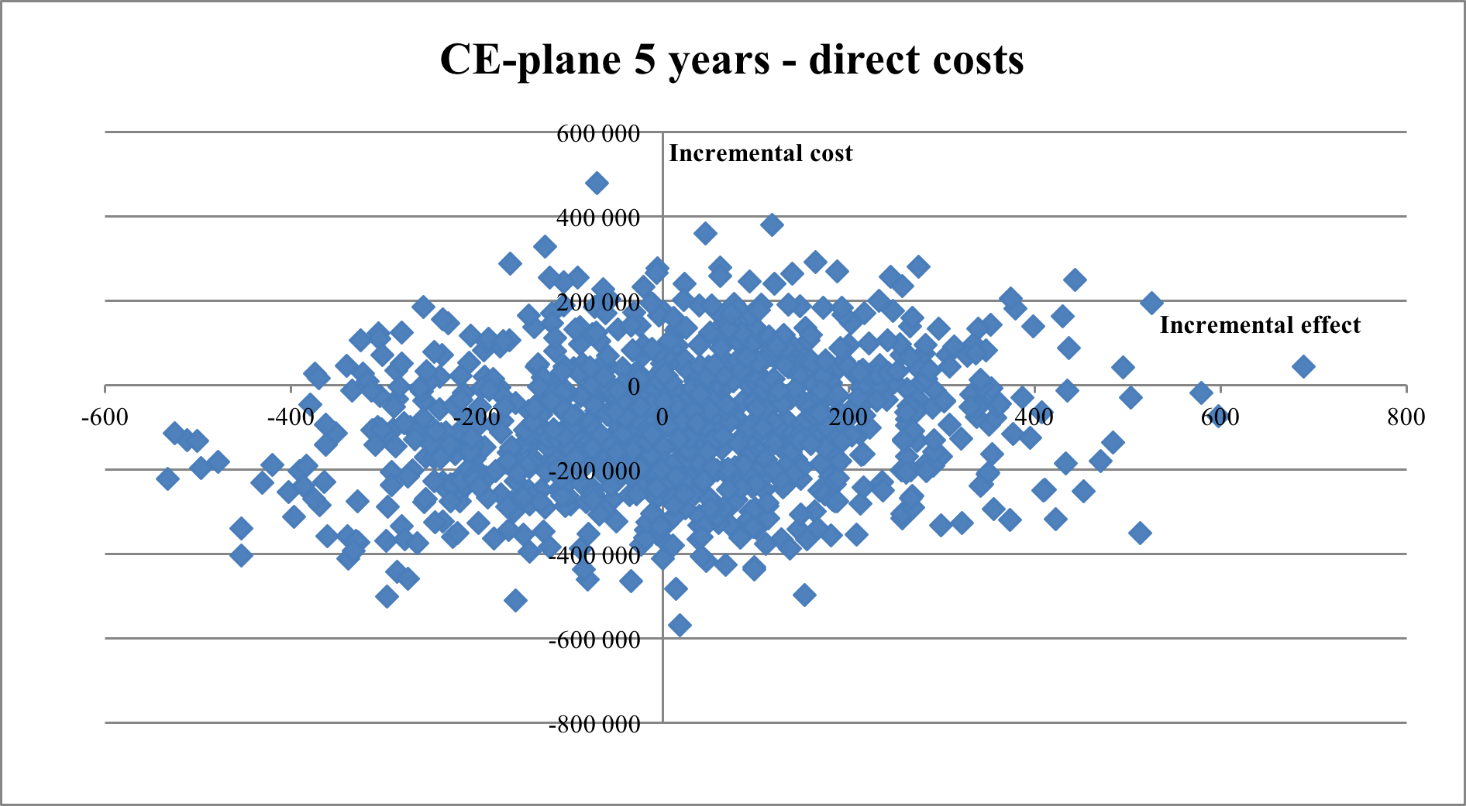


**Fig. S8** Cost-effectiveness acceptability curve (CEAC) illustrating the likelihood that the incremental cost-effectiveness ratio falls below a given threshold. A five-year time perspective and healthcare perspective


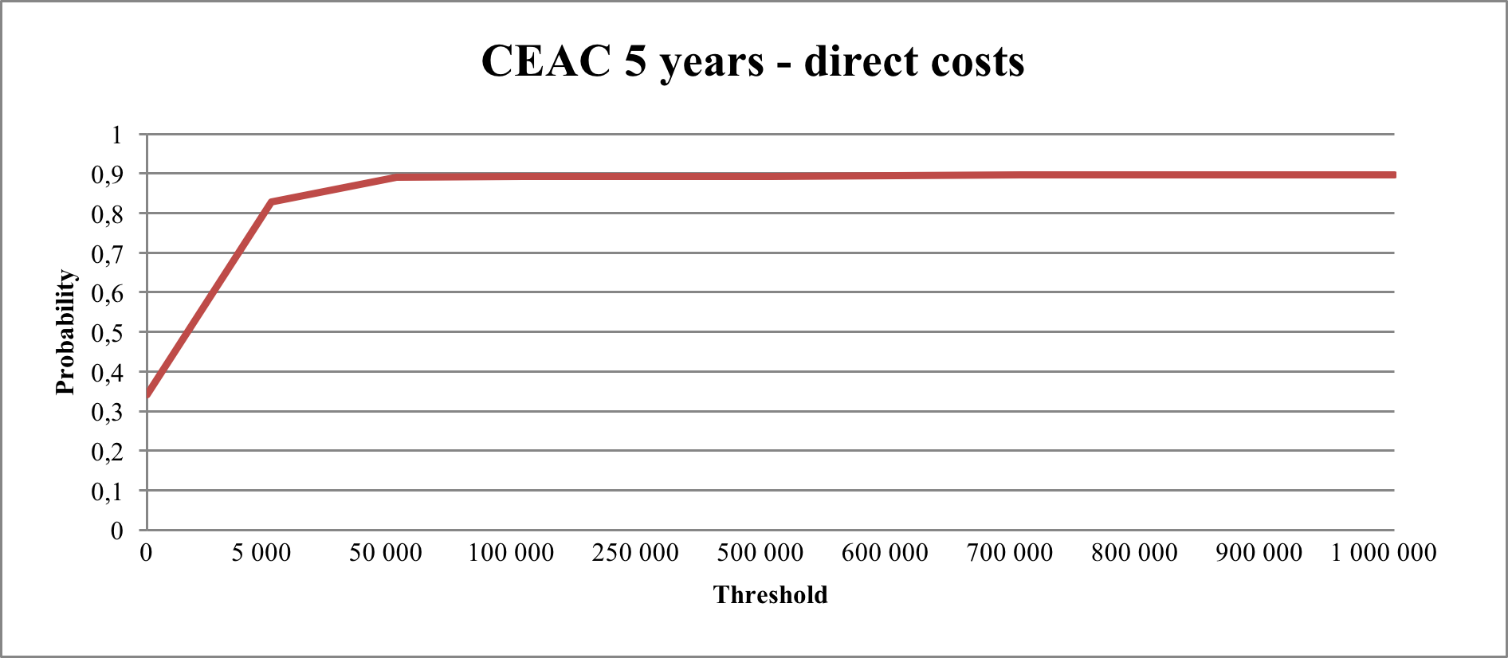


*Deterministic sensitivity analysis*

Results from the deterministic sensitivity analysis (2) - (6) described in the main text are illustrated in the figures S9-S13 below. Each parameter is allowed to increase from the base-case value to values where the ICER is both under and over the informal willingness-to-pay threshold per QALY in Sweden (500 000 SEK/QALY).

**Fig. S9** Sensitivity analysis for risk of first relapse in the person-centred care group. The figure plots the incremental cost-effectiveness ratio against the risk, for a range of risks enclosing the SEK 500 000 per additional QALY willingness to pay. The lowest risk (red data point) is the risk used in the base-case calculations

**
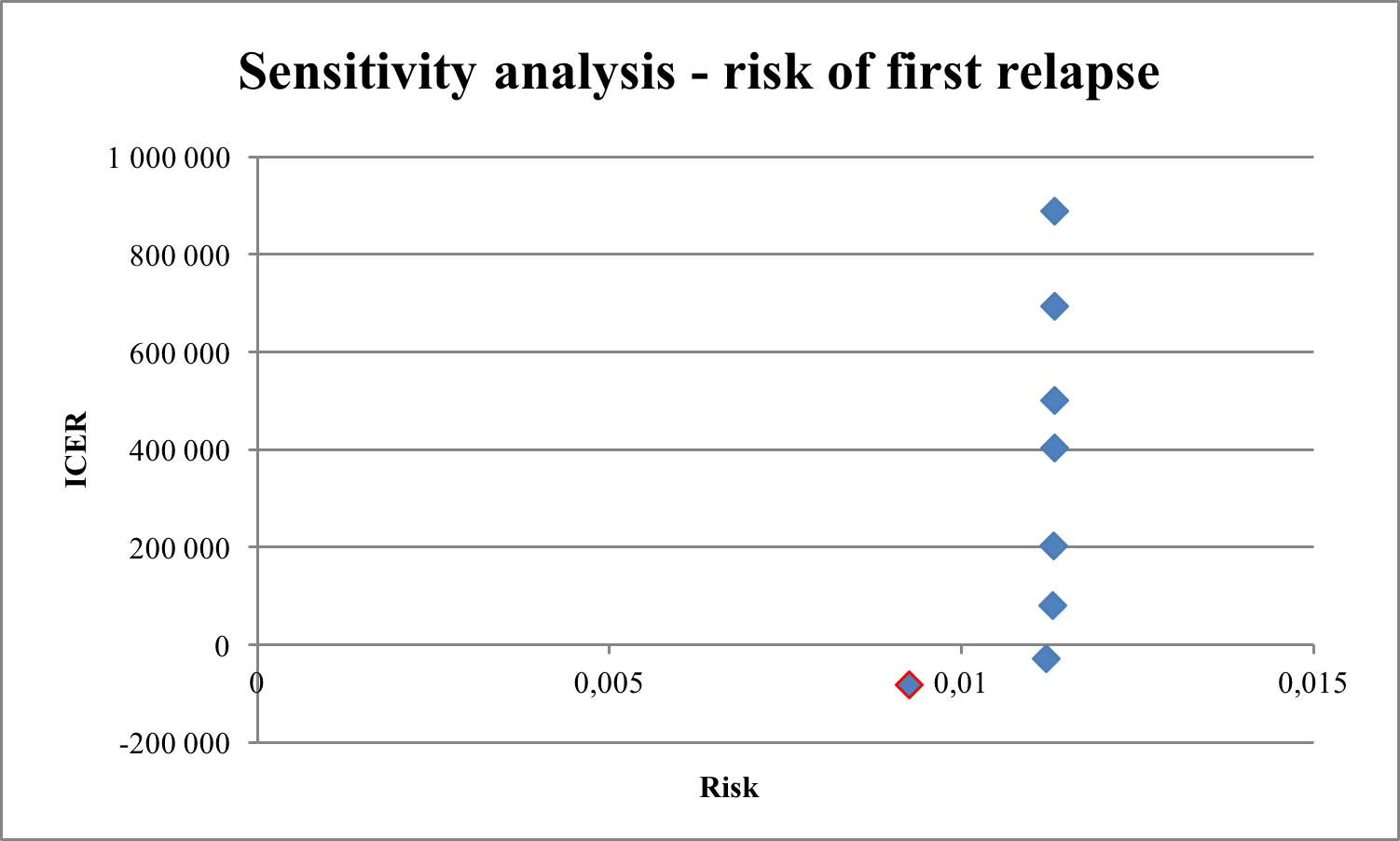
**

**Fig. S10** Sensitivity analysis for risk of second relapse in the person-centred care group. The figure plots the incremental cost-effectiveness ratio against the risk, for a range of risks enclosing the SEK 500 000 per additional QALY willingness to pay. The lowest risk (red data point) is the risk used in the base-case calculations

**
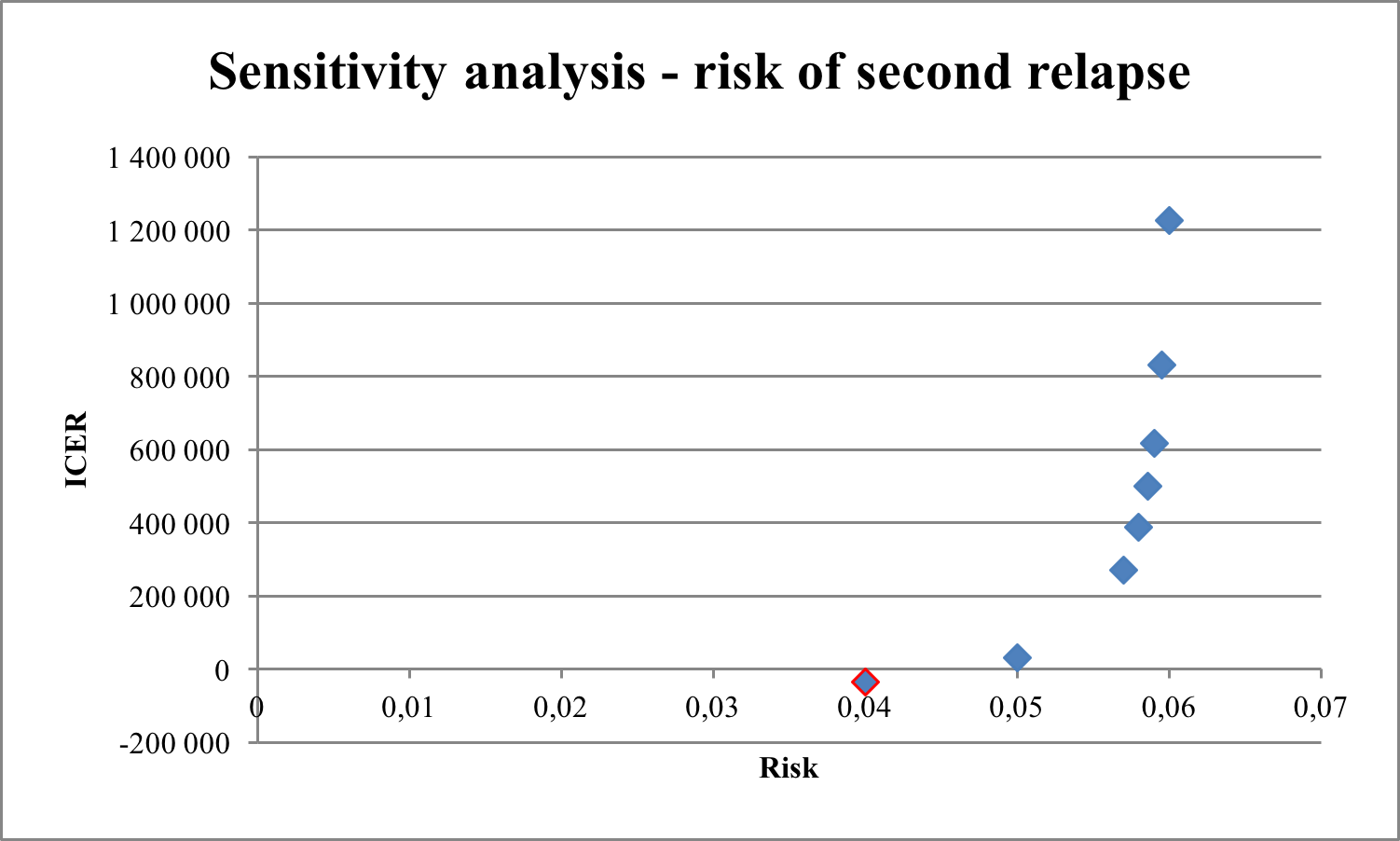
**

**Fig. S11** Sensitivity analysis for monthly indirect costs due to sickness absenteeism in the person-centred care group. The figure plots the incremental cost-effectiveness ratio against the indirect costs, for a range of costs enclosing the SEK 500 000 per additional QALY willingness to pay. The lowest cost (red data point) is the cost used in the base-case calculations


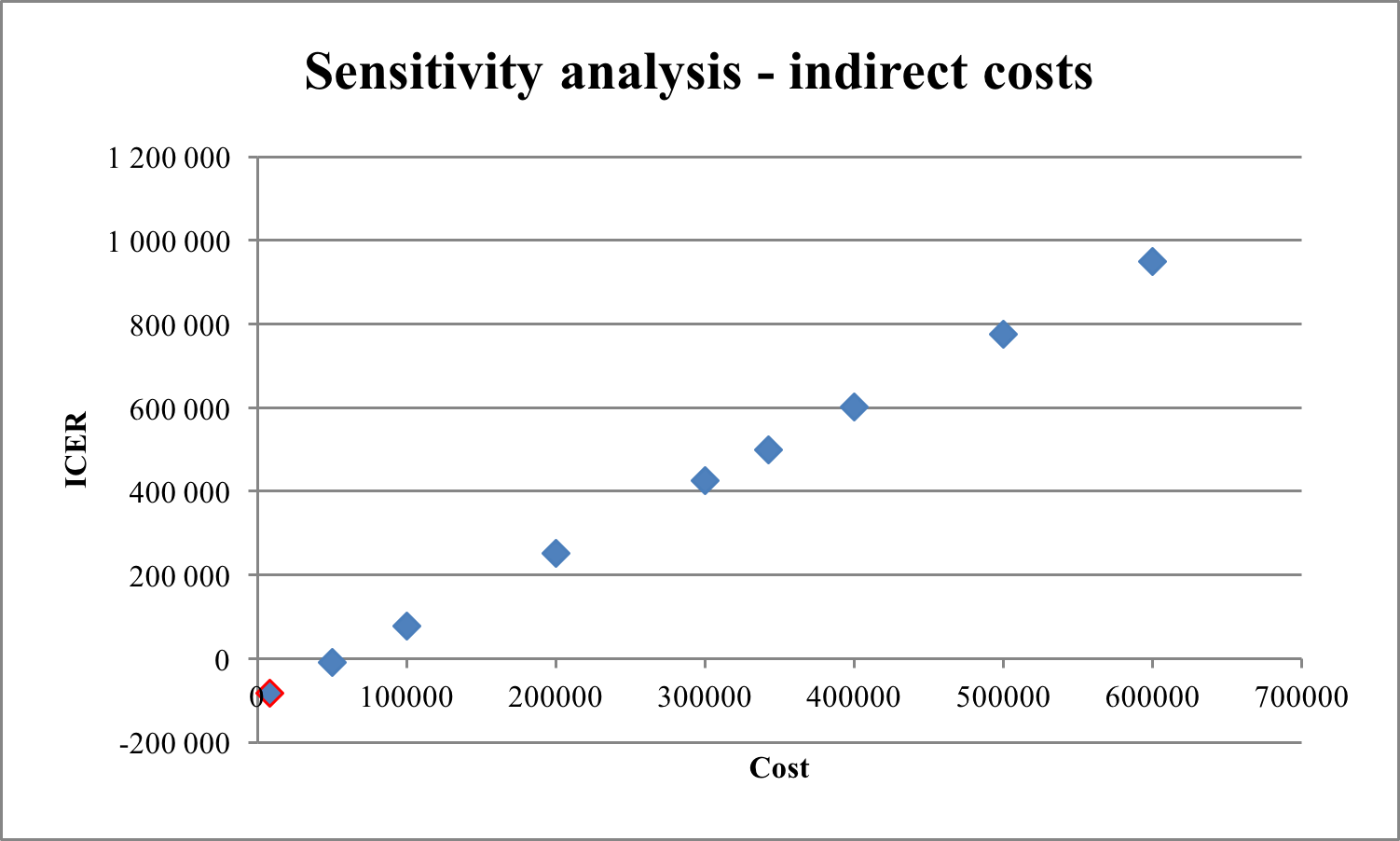


**Fig. S12** Sensitivity analysis for costs in remission in the person-centred care group. The figure plots the incremental cost-effectiveness ratio against the monthly healthcare cost in remission, for a range of costs enclosing the SEK 500 000 per additional QALY willingness to pay. The lowest cost (red data point) is the cost used in the base-case calculations


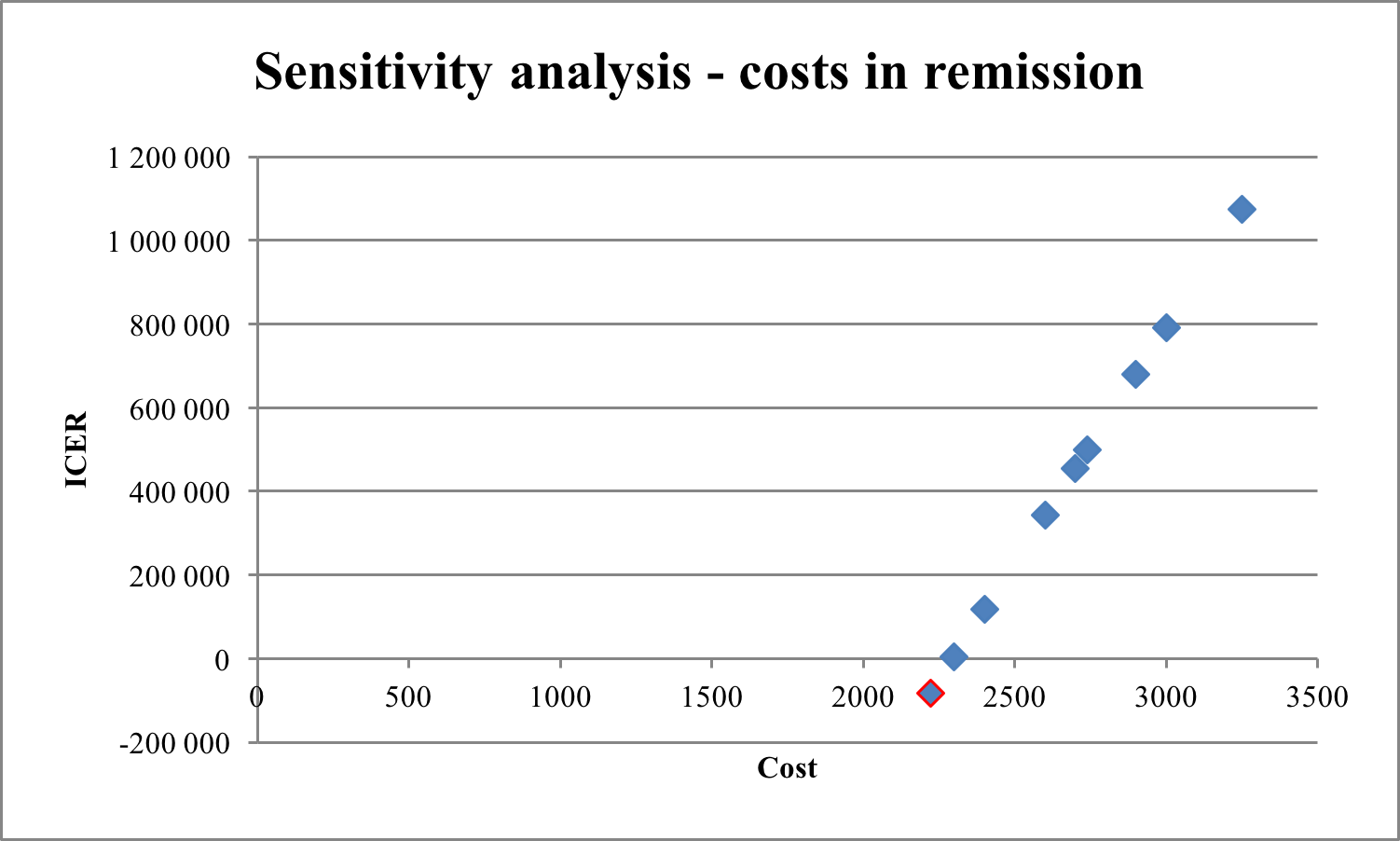


**Fig. S13** Sensitivity analysis for costs in relapse in the person-centred care group. The figure plots the incremental cost-effectiveness ratio against the cost in relapse, for a range of costs enclosing the SEK 500 000 per additional QALY willingness to pay. The lowest cost (red data point) is the cost used in the base-case calculations


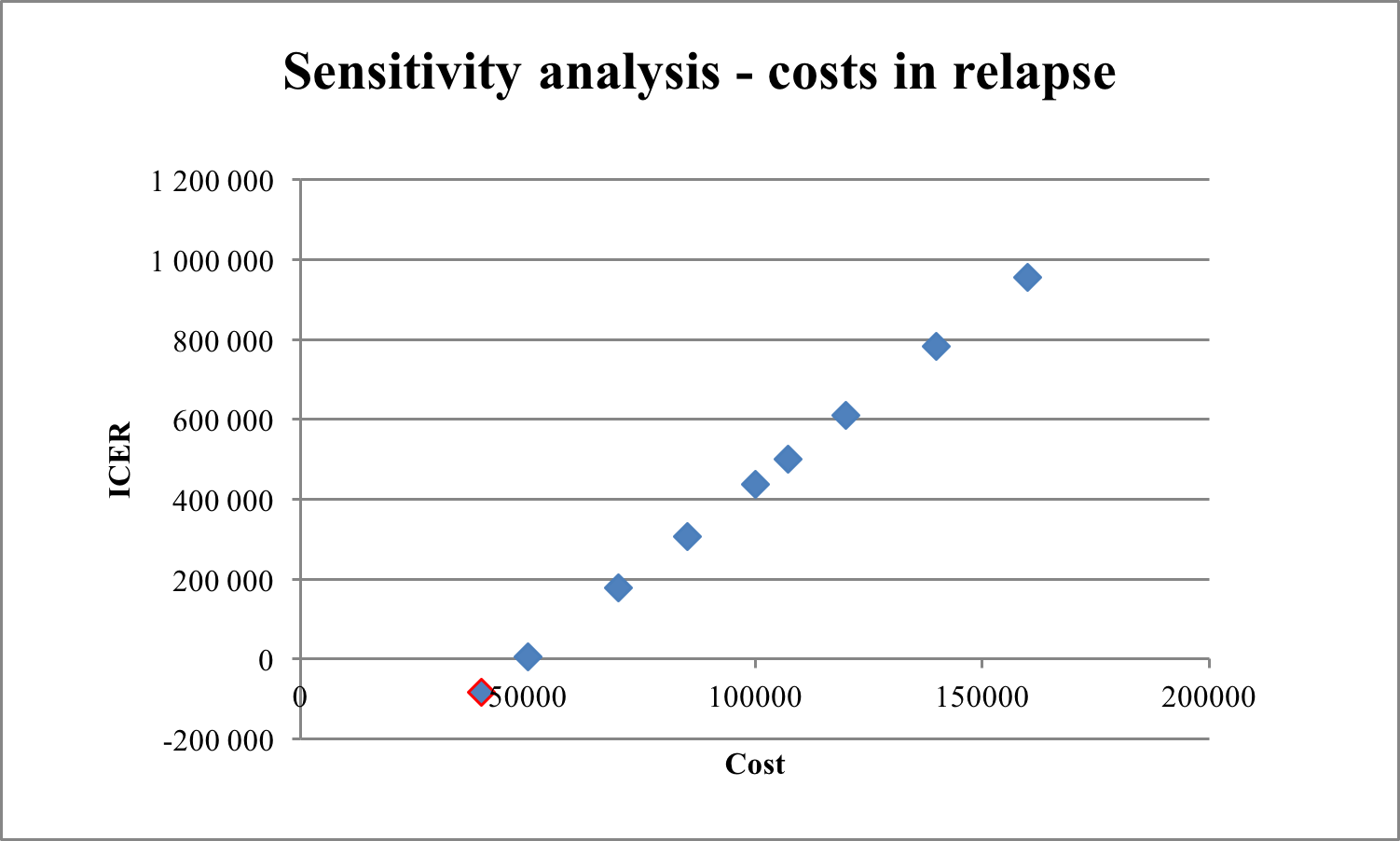

Supplement: Supplementary file 1 — Supplementary file1 (DOCX 1780 kb) [file 10198_2020_1230_MOESM1_ESM.docx]
